# Development and Implementation of a Nurse-Led Model of Care Coordination to Provide Health-Sector Continuity of Care for People With Multimorbidity: Protocol for a Mixed Methods Study

**DOI:** 10.2196/15006

**Published:** 2019-12-09

**Authors:** Kate M Davis, Marion C Eckert, Sepehr Shakib, Joanne Harmon, Amanda D Hutchinson, Greg Sharplin, Gillian E Caughey

**Affiliations:** 1 Rosemary Bryant AO Research Centre School of Nursing and Midwifery University of South Australia Adelaide Australia; 2 Discipline of Pharmacology, Adelaide Medical School Faculty of Health Science University of Adelaide Adelaide Australia; 3 School of Pharmacy and Medical Sciences University of South Australia Adelaide Australia; 4 School of Nursing and Midwifery University of South Australia Adelaide Australia; 5 School of Psychology, Social Work, and Social Policy University of South Australia Adelaide Australia; 6 Clinical Pharmacology Royal Adelaide Hospital, North Terrace Adelaide Australia

**Keywords:** continuity of patient care, multimorbidity, nurse led, integrated health, transitional, chronic disease

## Abstract

**Background:**

Innovative strategies are required to reduce care fragmentation for people with multimorbidity. Coordinated models of health care delivery need to be adopted to deliver consumer-centered continuity of care. Nurse-led services have emerged over the past 20 years as evidence-based structured models of care delivery, providing a range of positive and coordinated health care outcomes. Although nurse-led services are effective in a range of clinical settings, strategies to improve continuity of care across the secondary and primary health care sectors for people with multimorbidity have not been examined.

**Objective:**

To implement a nurse-led model of care coordination from a multidisciplinary outpatient setting and provide continuity of care between the secondary and primary health care sectors for people with multimorbidity.

**Methods:**

This action research mixed methods study will have two phases. Phase 1 includes a systematic review, stakeholder forums, and validation workshop to collaboratively develop a model of care for a nurse-led care coordination service. Phase 2, through a series of iterative action research cycles, will implement a nurse-led model of care coordination in a multidisciplinary outpatient setting. Three to five iterative action research cycles will allow the model to be refined and further developed with multiple data collection points throughout.

**Results:**

Pilot implementation of the model of care coordination commenced in October 2018. Formal study recruitment commenced in May 2019 and the intervention and follow-up phases are ongoing. The results of the data analysis are expected to be available by March 2020.

**Conclusions:**

Nursing, clinician, and patient outcomes and experiences with the nurse-led model of care coordination will provide a template to improve continuity of care between the secondary and primary health care systems. The model template may provide a future pathway for implementation of nurse-led services both nationally and internationally.

**International Registered Report Identifier (IRRID):**

DERR1-10.2196/15006

## Introduction

### Background

Increasing prevalence and complexity of multimorbidity across populations is a global phenomenon [1-3]. This constitutes one of the most significant challenges for health care in the 21st century [4]. In general, care for patients with multimorbidity is fragmented and not coordinated, especially between health care settings, such as primary and secondary care [5,6]. Current models of care delivery focus on single-disease-specific management, resulting in attendance at multiple specialist medical clinics, and do not support continuity of care, placing a significant burden on both patients and hospitals [3,7].

In an attempt to improve efficiencies within health care, nurse-led clinics and services have emerged over the past two decades [8,9]. Their effectiveness has been demonstrated on a number of levels, responding to the complexity of care coordination required by patients [10,11]. This includes the use of a person-centered approach [9], positive patient experience and satisfaction [9,12], and counseling and interventions to support chronic medication adherence [8,13].

In Australia, providing continuity in health care for people with chronic and complex disease is problematic, partly due to differences between federal and state government policies as well as structures and funding systems for the primary and secondary health care sectors [14]. This issue poses a challenge for nurse-led services to provide integrated models of care and lead continuity of care strategies between the health sectors at local service levels. Nurse-led models of care can provide a solution, in part, to the barriers associated with developing nonfragmented care in order to provide effective management for people with multimorbidity. It is, therefore, timely that a model of care trialing cross-sector collaboration is implemented. In Australia, the primary and secondary health care sectors will have congruent access to patients’ health care information through national strategies, such as My Health Record [15] and Health Care Homes [16]. These strategies, although in their infancy, if supported at local service and health network levels, can be used to leverage communication and collaboration by nurses to improve continuity of care. However, there remain few studies examining nurse-led models of care to improve continuity of care [17,18]. Despite the success of nurse-led services in a range of other contexts, their effectiveness in supporting continuity of care between the secondary and primary health sectors for people living with multimorbidity is yet to be determined.

### Multimorbidity and Nurse-Led Models of Care

Chronic diseases, including cardiovascular disease, diabetes, chronic lung disease, and cancer, are collectively responsible for almost 70% of all deaths worldwide. In the United Kingdom, the United States, and Australia, between 22% and 25% of the population live with multimorbidity, defined as having two or more chronic conditions concurrently; the prevalence of multimorbidity is even higher in the older population [2].

Multimorbidity is associated with poorer health outcomes, increased care fragmentation [1,19], higher health service utilization, and higher health care costs [20,21]. Existing models of care are based on a medical model of health service delivery and are designed to manage a single disease; therefore, they are not suitable for the complexity of health care associated with the presence of multiple chronic conditions [3,22,23]. Additionally, clinical guidelines predominantly focus on a single disease, potentially contributing to conflicting medication and care management for people with multimorbidity [24]. The traditional single-disease focus of current health care models and practices is also unsuitable for people with multimorbidity due to a lack of holistic care management and coordination [3,22,23].

Continuity of care is acknowledged as an essential component of high-quality care [25]. However, it is evident that chronic and complex health care management poses a challenge for health care systems to provide and promote continuity of care for people with multimorbidity [1]. A person-centered approach rather than a single-disease management program will provide more effective, high-quality care [4]. A coordinated comprehensive patient-centered model that focuses on continuity of care across the health system is especially needed for people with multimorbidity [6,26,27].

The relationship between aspects of continuity of care and patient satisfaction, improved health outcomes, a reduction in hospital admissions, and a reduction in health care utilization has been established [17,25,28,29]. In a recent scoping study [6] it was identified that in relation to multimorbidity management, models and elements of care were focused on general integrated care, as previously applied to single-disease management; therefore, they were unsuitable to the specific care required for the complexity associated with multimorbidity. The details of models of care require further study, specifically the role of nursing and nurse-led services to improve continuity of care and care coordination for people with multimorbidity.

## Methods

### Ethics and Registration

Ethical approval was obtained by the Human Research Ethics Committee (HREC) (reference number: HREC/17/RAH/552) at the University of South Australia (application ID: 200958) and the Central Adelaide Local Health Network (CALHN) (reference number: R20171204).

### Aims

The overall aim of this study is to determine the feasibility of implementing a nurse-led care coordination service from the outpatient setting to provide continuity of care across the secondary and primary health care settings for people with multimorbidity. The specific aims are as follows:

1. Develop and implement a model of care for a nurse-led service to provide continuity of health care for people with multimorbidity.
2. Identify nursing interventions associated with implementation of a nurse-led service model of care.
3. Identify barriers and enablers to implementing a nurse-led service.
4. Identify structures, processes, and outcomes required to implement a nurse-led service and achieve continuity of care.

### Design

#### Overview

The study design comprises action research with the application of the research spiral: “plan, act, observe, reflect, and re-plan” [30]. A Donabedian model [31] of evaluating structure, process, and outcome in health care will guide data collection during the action research cycles. There is precedence in the application of this model, not only in health care evaluation [32] but also in defining and evaluating nurse-led services [10,33]. The categories of structure, process, and outcome will include the measurement of stakeholder views and clinical staff and patient experience related to continuity of care across secondary and primary health care settings over time. The research will be conducted in two phases.

#### Phase 1: Initial Action Research Cycle

The goals of Phase 1 are as follows:

1. Consult with the Multidisciplinary Ambulatory Consulting Service (MACS) staff and associated stakeholders regarding the components and development of a nurse-led service model of care; for specific stakeholders, see Participants section and Table 1 below.
2. Review evidence in relation to nurse-led services, nursing interventions, and associations with continuity of care for people with chronic disease.
3. Review evidence in relation to best practice management of people with multimorbidity.
4. Collaboratively develop a nurse-led service model of care.
5. Develop operational roles, guidelines, and protocols to implement the nurse-led service model of care.

Phase 1, the initial action research cycle, will focus on two interventions. First, we will complete a systematic review to identify the effectiveness of nurse-led services to improve continuity of care for people with chronic disease (international prospective register of systematic reviews [PROSPERO] registration number: CRD42018095780). The second focus will be on stakeholder engagement; a series of forums, workshops, and meetings will engage stakeholders and collaboratively develop a model of nurse-led care coordination.

**Figure 1:**
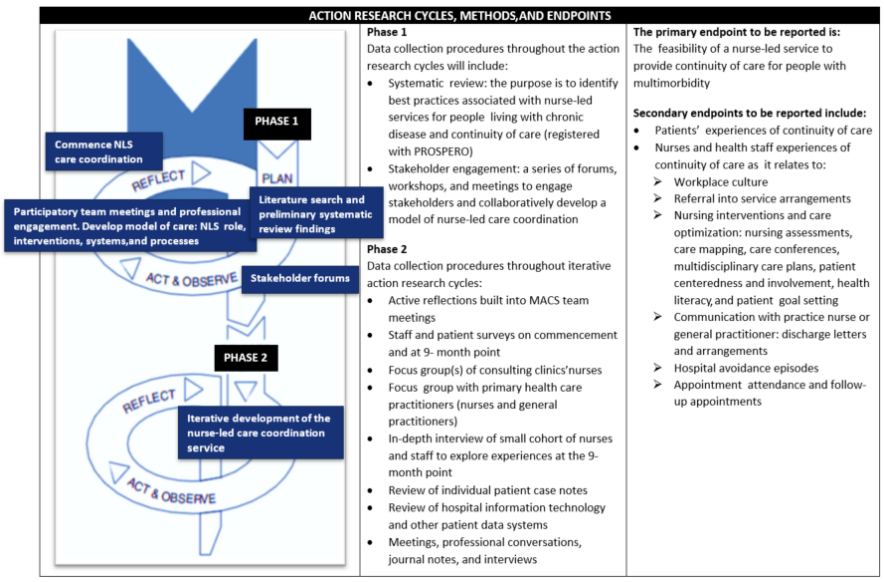
Design of the Multimorbidity Nursing Model of Care study. MACS: Multidisciplinary Ambulatory Consulting Service; NLS: nurse-led services; PROSEPERO: international prospective register of systematic reviews.

This action research cycle will inform the development of an evidence-based model of care for a nurse-led service and prepare the clinical team for nurse-led service implementation. Figure 1 depicts the Multimorbidity Nursing Model of Care action research study design; included is the systematic review and stakeholder forum informing development and planning of the nurse-led care coordination service and subsequent iterative implementation of the nurse-led service.

**Table 1:** Study participants and eligibility criteria.

Eligibility criteria	Stakeholders	Health care staff	Patients
Inclusion criteria	Attendees at the stakeholder forums and workshop; stakeholders include health care professionals, primary and secondary health care executives, relevant academic and clinical participants, and consumer representatives (n=40)	Health care staff from the tertiary referral center and outpatient service associated with implementing and/or working in, or in collaboration with, the nurse-led care coordination service or outpatient services (n=30) Health care staff associated with implementing and/or working in collaboration with the nurse-led care coordination service: from the primary health care sector (n=10)	All patients receiving care from registered nurses within the MACS^a^, the nurse-led care coordination service, attending a general practitioner service or the PHC^b^ sector associated with the MACS (n=30 in clinic) Patients who have previously attended multiple medical outpatient clinic appointments (n=100 postal surveys)
Exclusion criteria	Nil	Nil	Patients with cognitive impairment

^a^MACS: Multidisciplinary Ambulatory Consulting Service.

^b^PHC: primary health care.

#### Phase 2: Subsequent Action Research Cycles

The goals of Phase 2 are as follows:

1. Trial implementation of the nurse-led service model of care over a series of iterative action research cycles.
2. Implement nurse-led service care coordinator role.
3. Implement associated protocols and guidelines to operationalize the nurse-led service model of care.
4. Evaluate action research cycles in terms of changes to nursing interventions, service structures, processes, and outcomes.

Phase 2, the subsequent action research cycles, will employ a mixed-methods approach with multiple data collection points. During implementation of the nurse-led model of care coordination, nursing roles and interventions, service structures, processes, and outcomes will be observed, refined, and reimplemented. Patient, nurse, and health care staff experience as well as organizational culture impacts will be measured. The structures and processes within the nurse-led service will be evaluated by recognized data collection instruments that examine patient and health care staff experiences of continuity of care, patient-related quality of life, and staff experience of organizational culture (see Table 2 for data collection instruments and characteristics). Nursing roles, tasks, skills, and knowledge will also be evaluated (see Table 2).

**Table 2:** Data collection instruments and characteristics.

Author (publication year); instrument	Instrument primary purpose and adaptation; Continuity of care domain	Validation, reliability, and context for use	Number of items; Response options
Glasgow et al, (2005) [34]; Patient Assessment of Chronic Illness Care (PACIC) survey	A validated patient self-report instrument to assess the extent to which patients with chronic illness receive care that aligns with the Chronic Care Model. Measures care that is patient-centered, proactive, and planned and includes collaborative goal setting; Problem-solving and follow-up support	A practical instrument that is reliable and has face, construct, and concurrent validity	The PACIC consists of five scales and an overall summary score
MacColl Center for Health Care Innovation (2000) [35]; Assessment of Chronic Illness Care (ACIC V3.5) survey	The ACIC addresses the basic elements for improving chronic illness care at the community, organization, practice, and patient level―a*dapted for use in the MACS*^a^ *setting*; Relational, management, and informational continuity	Preliminary data indicate that the ACIC is responsive to changes that teams make in their systems and correlates well with other measures of productivity and system change	Seven dimensions—each dimension includes a number of items; Point value is attributed to a choice of four levels across each item
The EuroQol^b^ Group (1990) [36] and Herdman et al (2011) [37]; Patient EQ-5D^c^	The EQ-5D is a standardized measure of health status, applicable to a wide range of health conditions and treatments. Developed by the EuroQol Group, it provides a simple, generic measure of health for clinical and economic appraisal.	Widely validated and contextualized; translated into over 170 language versions	Five dimensions (each with three or five levels), 15 items, and cross-walk value sets available to convert three-item survey to meaningful value equivalent to five-item survey; Tick box and visual analog
Berglund CB et al (2015) [38]; Patient satisfaction and continuity of care	The survey was originally developed for the patient-physician outpatient encounter [39]. It proved to capture changes in patient satisfaction over time. It has since been adapted to capture the patient-nurse outpatient encounter; Relational, management, and informational continuity	No formal validity and reliability testing, however, item generation including the testing procedure provides sufficient content validity	12 multiple-choice items, including items concerning waiting time, continuity of care, length of visit, information, interpersonal manner, and fulfilment of expectations; 4-point scale from 1 (Not at all) to 4 (Very much)
Uijen AA et al (2011 [40] and 2012 [41]); Nijmegen Continuity Questionnaire (NCQ)	To measure continuity of care from the patients’ perspectives across primary and secondary care settings; Personal continuity, team continuity, and cross-boundary continuity	Internal consistency, content validity, structural validity, and construct validity	28 items in three subdomains; 5-point scale from 1 to 5
Stokes T et al (2005) [42]; General Practitioners’ Views on Continuity of Care survey	Measures the perceived importance of the types of continuity of care and doctor or practice characteristics that may influence attitudes toward personal continuity of care—*adapted for nurse-patient context*; Relational, management, and informational continuity	Good internal consistency (alpha=.78). The scale score correlated highly with the overall rating of the importance of personal continuity (*P*<.001)	25 items over four domains; 5-point scale from 1 to 5
Cameron KS et al (2011) [43]; Organizational Culture Assessment Instrument	Assesses six key dimensions of organizational culture: dominant characteristics of an organization, organizational leadership, management of employees, organizational glue, strategic emphasis, and criteria of success	Widely tested	Six dimensions with four alternatives (24 items); 4-point scale from A to D
Gardner G et al (2017) [44]; The Advanced Practice Nursing Role Delineation Questionnaire (APRD)	A self-assessment tool that provides a standardized understanding of advanced practice. It is designed to support health service planning, cross-discipline team development, and demonstration of achievement of practice at this level.	Evidence based	Five items: clinical care, optimizing health systems, education, research, and leadership; 5-point scale from 0 to 4

^a^MACS: Multidisciplinary Ambulatory Consulting Service.

^b^EuroQol: European Quality-of-Life Scale.

^c^EQ-5D: European Quality-of-Life Five-Dimension Scale.

### Setting

The setting for this study is an outpatient MACS at a large secondary, tertiary referral, hospital.

### Participants

1. Stakeholders. These will consist of attendees at the forums and workshop:
  - Representative health professionals from the MACS clinic.
  - Consumer representatives and advocates.
  - Representative leadership associated with the MACS clinic (ie, nursing and medical).
  - Representatives from the primary health network and private sectors (n=40).
2. Health care staff. There will be two health care staff groups:
  - Health care staff within the MACS or from the outpatient service (n=130).
  - Health care staff from the primary health network or the private sector. For example, clinical staff working in general or community settings (ie, primary health care sector) and have patients who attend or could attend the MACS clinic (n=10).
3. Patients. There will be two patient groups:
  - Patients who have previously attended the MACS clinic prior to implementation of the nurse-led care coordination service (n=100).
  - Patients who would usually attend the MACS clinic following implementation of the nurse-led care coordination service (n=30).

### Data Collection and Analysis

This action research study is largely qualitative but includes a quantitative descriptive element. There will be an initial stakeholder forum to develop the domains for a model of nurse-led care coordination. When the model is developed, it will be validated through current literature and a follow-up-focused workshop of stakeholders (see Table 3). Stakeholders at the forum and workshop will include health care staff and executives from both the primary and secondary heath care sectors, as well as other relevant academic and clinical participants (see Table 1). The nurse-led model for care coordination will then be implemented and refined through a series of iterative action research cycles. Data will be collected throughout the action research cycles (see Table 3).

**Table 3:** Data collection and analysis: survey and interview schedule.

Event and survey tool	Data collection point	Participants	Analysis
Stakeholder forums and validation workshop: activities guided by the Australian Primary Health Care Nurses Association, Building Blocks [45], and Donabedian’s categories of structure, process, and outcome [31]	At stakeholder forums and validation workshop events	Key stakeholders: registered nurses (level one), nursing middle management, general practitioners, pharmacists, allied health, and executives across both primary and secondary heath care sectors, along with consumer, academic, and professional association representation (n=60)	Thematic analysis
Patient Assessment of Chronic Illness Care (PACIC) survey [34]	Prior to nurse-led service implementation, January-April 2019	MACS^a^ outpatients who attended clinic prior to model of nurse-led care coordination implementation (n=100)	Descriptive statistics and thematic analysis
Assessment of Chronic Illness Care (ACIC V3.5) survey [35]	Prior to nurse-led service implementation, January-April 2019	MACS outpatients who attended clinic prior to model of nurse-led care coordination implementation (n=100)	Descriptive statistics and thematic analysis
Patient experience and continuity of care in clinics survey [38]	First appointment	MACS outpatients who attended clinic after model of nurse-led care coordination implementation (n=30-40)	Descriptive statistics and thematic analysis
Patient EQ-5D-3L^b^ health questionnaire [36,37]	At first and second appointments	MACS outpatients who attended clinic after model of nurse-led care coordination implementation (n=30-40)	Descriptive statistics
Patient experience and continuity of care in clinics, Nijmegen Continuity Questionnaire (NCQ) [40,41]	At second appointment or at 3-6 months	MACS outpatients who attended clinic after model of nurse-led care coordination implementation (n=30-40)	Descriptive statistics and thematic analysis
General Practitioners’ Views on Continuity of Care survey [42,46]	At commencement, then at 3-6 months	Nurses working in the MACS outpatient clinic (n=2)	Descriptive statistics and thematic analysis
Doctor and allied health staff experience and continuity of care survey [42,46]	At commencement, then at 3-6 months	Doctors and allied health staff working in the MACS outpatient clinic (n=3-10)	Descriptive statistics and thematic analysis
Primary health care staff experience and continuity of care survey [42,46]	At commencement, then at 3-6 months	Health care staff managing MACS patients in the primary health care sector; general practitioner rooms or community services (n=10)	Descriptive statistics and thematic analysis
Nurse experience and continuity of care survey, other than MACS [42,46]	At commencement	Nurses, other than the MACS nurses, working in outpatient clinics (n=80)	Descriptive statistics and thematic analysis
The Advanced Practice Nursing Role Delineation Questionnaire (APRD) [44]	At commencement and at 6 months	Nurses working in the MACS clinic and outpatient clinic nurses (n=2)	Descriptive statistics and thematic analysis
Staff workplace culture survey [43]	Commencement and at 6 months	All health care staff working in the MACS outpatient clinic (n=5-10)	Descriptive statistics and thematic analysis
Survey: question bank	At 6 months via email	Director of nursing and nursing director (n=2)	Thematic analysis
Interview, with questions from bank, and ongoing reflective meetings	At 6 months and ongoing	Head of unit (n=1)	Thematic analysis
Interview and ongoing reflective meetings	At 6 months and ongoing	MACS nurses (n=2)	Thematic analysis
Interview and ongoing reflective meetings	At 6 months and ongoing	MACS team (n=5-10)	Thematic analysis
Focus group questions from bank	At 6 months	Consulting clinics nurses (n=10-20)	Thematic analysis
Patient medical record	Following patient recruitment	MACS outpatients who attended clinic after model of nurse-led care coordination implementation (n=30-40)	Descriptive statistics

^a^MACS: Multidisciplinary Ambulatory Consulting Service.

^b^EQ-5D-3L: European Quality-of-Life Five-Dimension Three-Level Scale.

### Qualitative Data

Thematic analysis based on the phases of Braun and Clarke [47] will be used as outlined in Table 4. The process of thematic analysis will identify categories of information and develop model domains for developing a model of nurse-led care coordination. It is anticipated that the model will be pragmatic and consider specific continuity of care strategies to be implemented as part of the nurse-led care coordination service. Thematic analysis will also be used to analyze survey and interview data to reveal patients’, nurses’, and health care staff’s experiences of continuity of care, before and after implementation of the nurse-led model of care coordination. Through the process of thematic analysis, an account of what is happening in the situation (ie, nurse-led service within the multidisciplinary MACS clinic) and how it is happening will be identified [47]. Braun and Clarke’s practical approach is useful for comparing multiple data sources (ie, from patients, nurses, and health care staff) [47,48].

**Table 4:** Process of thematic analysis, adapted from Braun and Clarke [47].

Phase		Activity
**Analysis**		
	Familiarization with data	Transcribe data and formulate ideas; analysis starts here and continues throughout the process
	Generation of initial codes	Systematically code and collate entire dataset
	Search for themes	Sort different codes into possible *candidate* themes
	Review of themes	Refine and finalize candidate themes
	Naming and defining of themes	Develop thematic map of data, further refine themes, and perform final analysis
Production of the report		Perform inductive thematic analysis, which will emphasize understanding the patients’ and nurses’ experience of the nurse-led service

### Quantitative Data

Quantitative data from questionnaires and/or medical records related to patients’, nurses’, and health care staff’s experiences of continuity of care and the nurse-led model of care coordination and demographic data, as well as data relating to the nurse-led model of care coordination, continuity of care, and patient progress or outcomes will be analyzed (see Table 3). Analysis will use descriptive statistical methods including means, medians, and interquartile ranges where appropriate. Differences between variables will be analyzed using either two-tailed *t* tests or the Wilcoxon ranked-sum test where appropriate. A *P* value of less than .05 will be considered statistically significant. All statistical analyses will be conducted using NVivo 10 (QSR International) and SPSS, version 25 (IBM Corp).

### Validity and Reliability

The nurse-led model will be collaboratively developed at a series of stakeholder—secondary and primary health sectors—forums. The forums will also be developed with reference to the Australian Primary Health Care Nurses Association Building Blocks for nurse-led clinics [45]. A follow-up validation workshop and literature search will further refine the model. Implementation of the model through iterative research cycles will continue the validation process as elements change in response to user experiences. Interventions aligned with the model will be based on real-world experience in the nurse-led service, a consensus approach, and systematic findings from the literature.

Recognized and validated instruments will be used to collect data in relation to continuity of care, patient centeredness, workplace culture, and the practice role and level of the nurses (see Table 2). A concurrent approach to data collection and analysis will allow the separate use of quantitative and qualitative methods within a single cycle of data collection and analysis. This will allow both sets of data to be interpreted together, providing a richer and more comprehensive response to research questions [49].

## Results

Pilot implementation of the model of care coordination commenced in October 2018. Formal study recruitment commenced in May 2019 and the intervention and follow-up phases are ongoing. The results of the data analysis are expected to be available by March 2020.

## Discussion

### Expected Results

Nurse-led services and clinics have been widely implemented in primary health care settings and, increasingly, in outpatient departments but not with the purpose of improving continuity of care between the two sectors. The evidence for nurse-led services to improve continuity of care for people living with multiple chronic diseases and complex care needs has not been established. This proposed study is significant because it aims to develop a model of care for nurse-led services, based on both research and stakeholder experience. This model, focusing on patient-centered care and the nursing role to coordinate care to achieve continuity across the health care sector, has not been previously trialed for people with multimorbidity.

It is anticipated that the model of care for a nurse-led care coordination service will support the implementation of continuity of care strategies. These strategies may include assessment of risk of hospital readmission; patient readiness for change; well-coordinated, individualized, multidisciplinary health care plans; patient self-management strategies; and coordinated communication between the secondary and primary health care sectors. Ideally, these will result in improved patient and staff experiences and health outcomes. Development of the model followed by a series of action research cycles of testing and refining the model will ensure that the research incorporates both theory and practical experience related to continuity of care across the health sector. This action research approach will, therefore, focus on what works within a *real-world* clinical setting. It will produce a patient-centered model of care for nurse-led services that provides a template for continuity of care, articulating the nursing role and service for adaptation throughout diverse health care systems within Australia and potentially worldwide.

### Limitations

As this is an action research design, there are no a priori design of the nurse-led model of care coordination or nursing interventions required. However, as both the model and the interventions will be developed in collaboration with *real-world* clinical practice and the health care literature, it will be important to ensure concordance between both. No control or comparator will be included within the model assessment, but survey participants who attended the outpatient department prior to commencement of the nurse-led service will be examined. The service setting is geographically limited since it is located at only one site; however, it is anticipated that the setting will be adaptable and applicable to geographically diverse locations. Electronic record and patient data systems vary across health sectors and can pose access and consistency issues. These will be addressed through the highly pragmatic nature of the study, which will focus on relationship building [47] and regular and consistent communication across health sectors as part of the nursing interventions.

## Abbreviations

ACIC: Assessment of Chronic Illness Care
APRD: The Advanced Practice Nursing Role Delineation Questionnaire
CALHN: Central Adelaide Local Health Network
EQ-5D: European Quality-of-Life Five-Dimension Scale
EQ-5D-3L: European Quality-of-Life Five-Dimension Three-Level Scale
EuroQol: European Quality-of-Life Scale
HREC: Human Research Ethics Committee
MACS: Multidisciplinary Ambulatory Consulting Service
NCQ: Nijmegen Continuity Questionnaire
PHC: primary health care
PROSPERO: international prospective register of systematic reviews
